# Reviewer agreement trends from four years of electronic submissions of conference abstract

**DOI:** 10.1186/1471-2288-6-14

**Published:** 2006-03-19

**Authors:** Brian H Rowe, Trevor L Strome, Carol Spooner, Sandra Blitz, Eric Grafstein, Andrew Worster

**Affiliations:** 1 Department of Emergency Medicine, University of Alberta. Edmonton, Canada; 2 Department of Public Health Sciences, University of Alberta. Edmonton, Canada; 3 e-Health Services, Winnipeg Regional Health Authority, Winnipeg, Canada; 4 Department of Emergency Medicine, St. Paul's Hospital, Providence Health Group, Vancouver, Canada; 5 Department of Emergency Medicine, Hamilton Health Sciences and Division of Emergency Medicine, McMaster University, Hamilton, Canada

## Abstract

**Background:**

The purpose of this study was to determine the inter-rater agreement between reviewers on the quality of abstract submissions to an annual national scientific meeting (Canadian Association of Emergency Physicians; CAEP) to identify factors associated with low agreement.

**Methods:**

All abstracts were submitted using an on-line system and assessed by three volunteer CAEP reviewers blinded to the abstracts' source. Reviewers used an on-line form specific for each type of study design to score abstracts based on nine criteria, each contributing from two to six points toward the total (maximum 24). The final score was determined to be the mean of the three reviewers' scores using Intraclass Correlation Coefficient (ICC).

**Results:**

495 Abstracts were received electronically during the four-year period, 2001 – 2004, increasing from 94 abstracts in 2001 to 165 in 2004. The mean score for submitted abstracts over the four years was 14.4 (95% CI: 14.1–14.6). While there was no significant difference between mean total scores over the four years (p = 0.23), the ICC increased from fair (0.36; 95% CI: 0.24–0.49) to moderate (0.59; 95% CI: 0.50–0.68). Reviewers agreed less on individual criteria than on the total score in general, and less on subjective than objective criteria.

**Conclusion:**

The correlation between reviewers' total scores suggests general recognition of "high quality" and "low quality" abstracts. Criteria based on the presence/absence of objective methodological parameters (i.e., blinding in a controlled clinical trial) resulted in higher inter-rater agreement than the more subjective and opinion-based criteria. In future abstract competitions, defining criteria more objectively so that reviewers can base their responses on empirical evidence may lead to increased consistency of scoring and, presumably, increased fairness to submitters.

## Background

There is a large body of valuable and high-quality research that is, for whatever reason, never published in full manuscript format[1,2]. In fact, a published abstract is often the only permanent "official" source of information on a research project[3]. Abstracts presented at the meetings of specialty and generalist societies are now recognized as an important component of the grey literature searched in many systematic approaches to literature reviews. Exclusion of grey literature in meta-analyses has been found to exaggerate estimates of intervention effectiveness by 12% on average[4]. Provided it is subject to the same methodological assessment, grey literature including abstracts can contribute to a more complete synthesis of information than is possible using published manuscripts alone[5].

The goal of abstract review is to screen for submissions that are acceptable for inclusion in a conference program (in contrast to manuscript review, in which the abstract is checked to ensure that the content of the full text is accurately represented). The ability to effectively identify high-quality abstracts worthy of acceptance, presentation, and possible publication, is based on the criteria used to rank submissions. The quality of an abstract is typically assessed through the expert judgment of two or more independent reviewers. The objective assessment of an abstract's quality is made more difficult, however, by evidence suggesting that reviewers' judgments vary widely when evaluating others' work[6], even when the reviewers are considered "expert" in the field.

Variability is inherent in the abstract review process because no gold standard exists for evaluating the peer review process. One suggested measure of reliability for a set of criteria is how well reviewers agree on whether an abstract should score well or poorly[6]. If experts or members of a peer group do not consistently agree on the quality of abstracts, one could argue that the review process is arbitrary and open to bias. Conversely, higher inter-rater agreement suggests a more reliable system that is less likely to be influenced by bias. It is possible, however, that rather than indicating increased fairness, higher inter-rater agreement simply indicates a systematic bias where all abstracts of a certain type are impacted negatively (or positively) by bias. The purpose of this investigation was to:

1) quantify the level of agreement between conference abstract reviewers using the CAEP review criteria on the quality of abstract submissions; and

2) identify factors that might result in low agreement.

## Methods

### Submission system overview

The Canadian Association of Emergency Physicians (CAEP) has utilized a web-based system for abstract submissions and reviews since its 2001 Annual Scientific Meeting. The overall system was conceived of and designed by the CAEP Research Committee (RC), and programmed by one of the authors (TS). The computerized system utilized for abstract submissions and reviews was "VS Review" published by VS Communications Inc. in Edmonton, Alberta.

Each year, a call for abstracts is published in a fall issue of the *Canadian Journal of Emergency Medicine*[7,8] and on the CAEP web site[9]. Submissions are primarily from CAEP members, but a small percentage of submitters reside outside Canada and/or are not CAEP members. Information collected about each abstract are: the text of the abstract; desired mode of presentation (i.e. oral or poster); study type (i.e., research design e.g., randomized controlled trial, retrospective chart review); category (i.e., conference theme or track into which submissions are grouped e.g., administration, clinical practice, and informatics); and presenting author information (i.e., name, status, and affiliation).

### Review system overview

Each submitted abstract is assigned three independent reviewers from the bank of volunteer CAEP reviewers. An administrator (CS) coordinates the review process and attempts to prevent conflict-of-interest situations during the reviewer assignment phase. Conflict-of-interest declarations on the reviewers' part are requested if such conflict is discovered during an abstract review. Each reviewer is assigned 15 to 20 abstracts per annual conference, and is blinded to the abstracts' author(s) and source. The median number of reviewers for the years 2001 to 2004 was 24. The pool of reviewers remained largely the same through the first three years; however, several new reviewers were added in 2004.

Scoring is based on nine criteria (Table 1). The CAEP evaluation criteria were loosely adapted from another Emergency Medicine paper-based system (SAEM) designed for the screening of abstracts, and were electronically implemented using the VS Review tool. The scoring criteria were collaboratively developed by members of the CAEP Research Committee with research expertise in the various investigation types. In essence, the criteria have been developed by the very experts who would be using them to evaluate abstracts. The criteria changed slightly for 2003 and 2004 based on reviewer feedback and suggestions to improve clarity and remove ambiguity from the criteria.

**Table 1:** Abstract review criteria group used, and maximum point values, in 2001–2002 and 2003–2004 conference abstract submissions.

**Criteria Group**	**2001, 2002**	**2003, 2004**	**Maximum Points**
Hypothesis/Objectives/Intent	X	X	2
Study Design/Verification Procedures	X	X	2
Methods I	X	X	2
Methods II		X	2
Statistics	X	X	2
Presentation	X	X	2
Originality		X	2
Impact	X		3
Impact		X	4
Overall Impression	X	X	6
Conclusion	X		2
Recommendation	X		3

X = utilized

The methodological and statistical criteria vary depending on the type of study reported in the abstract. For example, review criteria for statistics and methodology differ between randomized controlled trials and qualitative studies. During development of the system, content experts on each research design examined the criteria for face validity. Each of the nine criteria contributes a maximum value of two to six points to the total score. When reviewing submissions, reviewers are asked to assign a score for each criterion based on the descriptive text next to the "radio button" on the evaluation from. Table 2 shows the complete criteria set for the "Controlled Clinical Trial" study type.

**Table 2:** Sample review critiera for "Controlled Clinical Trial" submission.

**Criterion**	**Score**	**Description**
Hypothesis	0	Unstated
	1	Clear, but not detailed
	2	Clear, comprehensive
Study Design	0	Inappropriate design
	1	Acceptable design
	2	Superior design (Please note that if the best way to answer the study question is by a RCT, then a RCT gets 2 points)
Methods I	0	Non-randomized
	1	Pseudo-randomized (day of the week, flip coin, etc)
	2	Randomized (random numbers tables, etc)
Methods II	0	Unblinded and outcome measure that are unblinded.
	1	Unblinded or single-blinded with blinded outcome measures
	2	Double-blinded
Statistics	0	Inappropriate and poorly described statistical methods.
	1	Appropriate but poorly described or reported.
	2	Appropriate and well reported (p-values/confidence intervals).
Presentation	0	Unclear, poorly organized and not conforming to CAEP format.
	1	Unclear, poorly organized or not conforming to CAEP format.
	2	Clear, well organized and conforming to CAEP format
Originality	0	Repetition of previous work.
	1	Unique slant on a common problem.
	2	Cutting edge, novel approach.
Impact	0	Will make no difference in practice
	1	Repetition and with no unique features.
	2	Important outcome, but intervention may be difficult to implement in other settings.
	3	Important outcome, could change practice
	4	Important outcome, changes practice.
Overall Impression	0	Unacceptable
	1	Very poor
	2	Poor
	3	Acceptable
	4	Good
	5	Very good
	6	Outstanding

The maximum total score a reviewer can award an abstract is 24 points, and the minimum is zero. The final score used for ranking an abstract is the average of the three reviewers' total scores. Abstracts are assigned acceptance priority based on final score and mode of presentation.

Although other approaches to variance analysis are possible, the investigators considered three main sources of variance that impact reviewer agreement to be: 1) abstract effect; 2) reviewer effect; and 3) abstract-reviewer effect[10]. The abstract effect is the extent to which the quality and presentation of an abstract contributes to the abstract's total score in a review system. The reviewer effect is the tendency for a reviewer to consistently score higher or lower as compared to the other reviewers. The reviewer-abstract effect is a non-systematic positive or negative reaction a reviewer might have to a given abstract that results in the reviewer assigning a higher or lower score than would normally be assigned. For example, a preference for one topic over another, or even the way one abstract is presented compared to another, might lead to differential scoring on the part of a reviewer. The goals of the review methodology are to reduce variability in scoring by maximizing the abstract effect, minimizing the reviewer effect, and controlling the abstract-reviewer effect. This is done primarily through carefully crafted scoring criteria, blinded reviewing, and prevention of conflicts of interest.

### Analysis

This study is a retrospective analysis of an existing administrative database used for the on-line submission and review of CAEP Annual Scientific Meeting abstracts. SPSS Version 11 (SPSS Inc, Chicago, Illinois) was utilized for statistical analysis.

Inter-rater agreement was measured using the Intraclass Correlation Coefficient (ICC), which is commonly used to measure agreement between two or more raters. Computed ICC values range from -1 (perfect disagreement) to +1, which occurs when assessments are in perfect agreement[11,12]. As the ICC value approaches 1.0, the variability in scores between abstracts can be attributed to actual differences in the abstracts themselves[11]. In this study, each abstract was rated by three randomly-assigned reviewers, therefore, a one-way random-effects model for ICC calculations is the most appropriate[12,13]. Although there is no ubiquitous "goodness" ratings for ICC, values previously used in literature for ICC[14] (as well as for Kappa[15], another measure of rater agreement used for dichotomous and interval data) are: ICC <0.20 "slight agreement"; 0.21–0.40 "fair agreement"; 0.41–0.60 "moderate agreement"; 0.61–0.80 "substantial agreement"; >0.80 "almost perfect agreement".

Comparisons between means were conducted using one-way analysis-of-variance (ANOVA) and, where appropriate, summary measures are presented with 95% confidence intervals (95% CI).

## Results

Over the four-year period being studied, 495 abstracts were electronically submitted. The mean final score of all 495 abstracts was 14.4 ranging from 14.8 (95% CI: 14.3–15.4) in 2001 to 14.06 (95% CI: 13.5–14.6) in 2003 (Table 3). There is no statistical difference in the mean scores over the four years (F = 1.442, df = 3, p = 0.229). ICC values for total scores, (representing overall reviewer agreement on the "quality" of an abstract), from 2001 to 2004 were 0.36, 0.43, 0.59, and 0.42, respectively (Table 3).

**Table 3:** Annual reviewer agreement measured by ICC, and mean total scores of submitted abstracts.

**Year**	**ICC (95% CI)**	**Mean Total Score (95% CI)**
2001	0.36 (0.24–0.50)	14.8 (14.3–15.4)
2002	0.43 (0.32–0.53)	14.2 (13.7–14.7)
2003	0.59 (0.50–0.68)	14.1 (13.5–14.6)
2004	0.43 (0.33–0.52)	14.5 (14.1–14.9)

Table 4 illustrates the amount of agreement within the individual criteria themselves. When examining the consistency of reviewer agreement within individual criteria utilized over the four-year period, "Hypothesis" remained the most consistent (with a maximum variation in ICC of 0.03) between 2001 and 2004. The criteria experiencing the most variation over the four-year period is "Overall", with a variation in ICC of 0.20. Eight of the nine criteria experienced their peak reviewer agreement in 2003. There is a strong correlation (Pearson r = 0.91, p < 0.05) between the "Overall" criteria score and total score of an abstract.

**Table 4:** Criteria-level intraclass correlation coefficient and 95% confidence interval by year.

	**2001**		**2002**		**2003**		**2004**	
**Criteria**	**ICC**	**95% CI**	**ICC**	**95% CI**	**ICC**	**95% CI**	**ICC**	**95% CI**

Hypothesis	0.24	0.11–0.37	0.27	0.16–0.39	0.25	0.13–0.37	0.26	0.16–0.36
Study Design	0.15	0.03–0.29	0.20	0.09–0.32	0.30	0.18–0.42	0.23	0.13–0.33
Methods I	0.26	0.14–0.40	0.27	0.16–0.39	**0.57**	0.47–0.66	**0.44**	0.35–0.53
Methods II		-		-	**0.59**	0.50–0.68	**0.51**	0.43–0.60
Statistics	0.34	0.22–0.47	0.38	0.27–0.50	**0.43**	0.32–0.54	**0.41**	0.31–0.50
Presentation	0.16	0.04–0.29	0.17	0.06–0.28	0.34	0.22–0.46	0.17	0.08–0.27
Originality		-		-	0.25	0.14–0.37	0.20	0.10–0.30
Impact	0.22	0.10–0.36	0.24	0.12–0.36	0.34	0.22–0.46	0.32	0.22–0.42
Overall	0.31	0.18–0.44	0.30	0.19–0.42	**0.50**	0.39–0.60	0.30	0.20–0.40
Conclusion	0.24	0.11–0.37	0.18	0.07–0.29				
Recommendation	0.21	0.09–0.35	0.34	0.23–0.45				

NB: Bold highlights ICC values that are greater than 0.40 ("moderate agreement")

Comparing the Table 4 data from a year-by-year perspective reveals that no criteria-level ICCs exceeded 0.40 ("moderate agreement") in 2001 and 2002. Maximum ICCs between 2001 and 2002 range from 0.15 (Study Design, 2001) to 0.38 (Statistics, 2002); the 95% CI of several criteria ICC values do cross the 0.40 level, however. The 2003 criteria ICCs ranged from 0.25 (Hypothesis and Originality) to 0.59 (Methods II), and eight of the nine criteria experienced their highest ICC in this year. The 2004 criteria ICC range was 0.17 (Presentation) to 0.51 (Methods II). The "peak" in reviewer agreement occurring in 2003 does not appear to have fully carried over in 2004.

In order to rank criteria across years to compare which criteria resulted in the best reviewer agreement, the ICC scores were averaged. Because of the changes to the criteria in 2003, criteria were ranked in two blocks: 2001–2002 (Block 1) and 2003–2004 (Block 2). The top four criteria in terms of reviewer agreement based on the rankings for 2001–2002 were Statistics, Overall, Recommendation, and Methods. For 2003–2004, the top four criteria were Methods II, Methods I, Statistics, and Overall.

## Discussion

This study examined the reviewer agreement using an electronic abstract scoring system for an emergency medicine (EM) conference. From 495 abstracts submitted over 4 years, this system demonstrated several significant findings. First, agreement between reviewers on total score should be considered only "moderate" since the total score ICC was between 0.21 and 0.40 (fair) in 2001 and between 0.41 and 0.60 (moderate) in 2002, 2003, and 2004. Agreement values in terms of total score are at least as good as those reported by others in the literature, which are usually less than 0.40[16]. Second, there was even less agreement (ICC<0.40; "slight" agreement) between reviewers on individual abstract scoring criteria. This suggests that reviewers can disagree on the particular details of what constitutes a high-quality or poor-quality abstract yet still generally agree at the overall quality. Third, better agreement is demonstrated in the more specific and objective criteria, which is in concordance with the literature[17]. The top-ranked criteria for reviewer agreement are ones that describe aspects of an abstract that are easier to rate objectively than the other criteria. For example, the Statistics criterion rates abstracts on whether appropriate statistics were utilized and on the presence or absence of reported confidence intervals and p-values. As well, the Methods I, Methods II, and Statistics (in addition to Study Design) change in accordance with the type of investigation represented by the abstract and chosen by the author, so they are more tailored to the exact requirements of the abstract. Finally, the "Overall" assessment by reviewers reached high levels of agreement and there was a strong correlation between the Overall criteria and the total abstract score.

The ICC for Methods I increased substantially for 2003 and 2004 as compared to 2001 and 2002 despite very similar review criteria. A possible reason for this is the way in which submission types are assigned and the fact that review criteria are tailored to each submission type. In 2001 and 2002, each reviewer selected the submission type (i.e., "survey") upon which the review criteria were based. Reviewers did not always agree on the type of submission type, however. If there was a mismatch in the submission types selected by the reviewers, the abstract would be rated on different Methods criteria. Starting in 2003, the CAEP Research Committee requested that authors select their submission type so the same Methods criteria would be utilized by all three reviewers. The Committee felt that this approach would increase fairness for submitters in that all reviewers would be judging by the same criteria. In 2003 and later, if an author selected an inappropriate submission type (i.e., "controlled clinical trial" instead of "survey"), the abstract lost points on Methods, but the loss was reflected consistently across reviewers and could not be attributed to bias on the part of reviewers.

Reviewer agreement achieved its maximum amount in the 2003 review season. This is likely due to the fact that the criteria were clarified and made less ambiguous (as a result of reviewer feedback), as well as due to the growing experience (and competence) of the pool of regular CAEP abstract reviewers. It is possible that the "peak" in reviewer agreement did not fully carry over in 2004 due to the addition of several new reviewers to the reviewer pool who would not have been as experienced in using the system to review and score abstracts.

As this project analyses an administrative process, there are limitations that lead to difficulty in generating meaningful analysis of the data. First, we have no prospective data on reviewer satisfaction or problems with interpretation. Second, a limited pool of reviewers were available from a small community of EM researchers in Canada, complicating the process of assigning reviewers in a random manner while avoiding conflicts. Considerable efforts were made to maintain blinding; we suspect, however, that total anonymity of the authors and blinding of reviewers to abstracts was impossible given the small EM research community. Third, reviewers were not necessarily assigned abstracts whose topics fell within their specialty or areas of expertise. This would perhaps limit a reviewer's ability to judge abstracts on some of the more technical criteria. In addition to these, there were other factors, including press deadlines, which precluded fully "scientific" assignment of reviewers to abstracts. Finally, one could argue that reviewer agreement is higher for simple methodological descriptors such as "double blinding" because this is based on the presence or absence of words in the abstract, not on a more complex evaluation of quality.

Notwithstanding the above concerns, this is one of the largest and most comprehensive evaluations of an electronic abstract scoring system ever conducted. This investigation, and the work of others, suggests ways in which the abstract review process can be improved upon for future years. The higher agreement between reviewers on the objective criteria suggests that modifications to the scoring sheets to reduce weighting of subjective criteria and increase weighting on the more objective criteria would further enhance scoring reliability. As well, modifying subjective criteria to incorporate less ambiguous guidelines or parameters might further improve reviewer agreement. Although the ICC one-way random effects model is not adversely affected by reviewer variability in scoring, it is nonetheless a good benchmark for agreement between reviewers. The tendency for some reviewers to consistently score higher (or lower) than others, the impact on overall reviewer agreement, and statistical methods to detect bias (and enhance overall fairness of reviews), need to be further investigated.

## Conclusion

Results from this investigation suggest that over the last four years the on-line conference abstract submission and peer review system developed by CEAP has generated moderate agreement among reviewers regarding the overall quality of submitted abstracts when the total score is used. Reviewer agreement declines somewhat when compared at an individual criterion level. Criteria that are based on empirical evidence and are less subjective appear to result in higher agreement. Differences of opinion will always occur; the responsibility of scientists is to determine when that difference in opinion adversely impacts scientific objectivity.

## Competing interests

Mr. Strome is a part-owner of the company that developed the review software (VS Communications Inc). No other author declares a conflict of interest.

## Authors' contributions

BHR is the lead author and contributed to developing the review criteria, the study design, manuscript preparation, and manuscript revision. TS developed the software, and contributed to the study design, statistical analyses, manuscript preparation, and manuscript revision. CS contributed to the study design and edited the manuscript. SB participated in the design of the study, statistical analysis, and edited the manuscript. EG and AW contributed to the study design and manuscript preparation.

## Pre-publication history

The pre-publication history for this paper can be accessed here:


